# Energy Costs of 37 Physical Activities in Chinese Children and Adolescents Aged 9–17 Years with Obesity

**DOI:** 10.3390/nu16223966

**Published:** 2024-11-20

**Authors:** Lin Zhu, Zekai Chen, Jingxin Liu, Youxiang Cao, Jing Liao, Weijun Xie

**Affiliations:** 1 School of Sport and Health, Guangzhou Sport University, Guangzhou 510500, China; zekaichen1993@163.com (Z.C.); 51245@gzsport.edu.cn (J.L.); 18520592540@163.com (W.X.); 2 Guangdong Provincial Key Laboratory of Physical Activity and Health Promotion, Guangzhou Sport University, Guangzhou 510500, China; 3 Key Laboratory for Exercise and Adolescent Physical Health, Guangzhou Sport University, Guangzhou 510500, China; 4 School of Physical Education and Sports, Soochow University, Suzhou 215021, China; liujingxin@suda.edu.cn; 5 School of Physical Education, Nanjing Xiaozhuang University, Nanjing 211171, China; caoyouxiang@njxzc.edu.cn

**Keywords:** energy expenditure, MET, obesity, exercise, youth

## Abstract

Background/Objective: Obtaining data on the energy expenditure of physical activity (PA) in children and adolescents with obesity is crucial for promoting health in this population through exercise. However, relevant research is limited. This study aimed to determine the energy expenditure characteristics of 37 diverse PAs in children and adolescents with obesity and examine the association between obesity and energy expenditure (EE) characteristics within this cohort. Methods: Three hundred and ninety-seven children and adolescents with obesity aged 9–17 performed various PAs. PA intensity, EE, and body fat (BF) percentage were measured. Results: The resting oxygen uptake (VO_2_) and resting energy expenditure (REE) of children and adolescents with obesity were 4.23 (3.72–4.84) ml/kg/min and 0.020 (0.018–0.023) kcal/kg/min, respectively, with significant differences between boys and girls (*p* < 0.05). The metabolic equivalent of task (MET) values for the PAs ranged from 1.12 to 8.14 METs. Regression analyses revealed an inverse association with age, BMI, and percentage BF increased, with resting VO_2_, REE, and average PA energy expenditure (PAEE) during PAs. Conclusions: (1) Resting VO_2_ was lower in children and adolescents with obesity than those with normal weight and higher than in adults. (2) Of the measured PA intensities, 8 were light, 17 were moderate, and 5 were vigorous intensity. (3) The increase in the degree of obesity and age further decreased VO_2_ and EE both at rest and during PA. Our results can provide precise guidance for the exercise of children and adolescents with obesity and serve as a reference for the development of physical activity guidelines and compendiums. Future research should further diversify the types of PAs measured.

## 1. Introduction

Obesity is related to multiple metabolic disorders and is a significant global public health problem in the 21st century [1]. In 2022, the World Health Organization reported that 2.5 billion adults aged 18 years and older were overweight, and 890 million were obese. Among children and adolescents aged 5–19 years, over 390 million were overweight, and 160 million were obese. Further, 27 million children under age 5 were overweight. Nearly half of young children who are overweight and obese live in Asia. The incidence of overweight and obesity in Chinese children and adolescents is increasing steadily. According to the *2021 Children’s Blue Book: China Children’s Development Report*, in 2010, 15.5% of Chinese school-age children were overweight and obese and in 2019, 24.2% were overweight and obese. By 2024, it was predicted that 29.4% of children and adolescents would be overweight and obese [2].

Overweight and obesity in children and adolescents affect physical and psychological health, growth and development, and metabolic health. Increased physical and metabolic risks include type 2 diabetes, cardiovascular diseases, bronchial asthma, obstructive sleep apnea, pediatric liver disease, hypertension, and gastroesophageal reflux [3]. The results from a meta-analysis of 15 prospective cohort studies reported that children and adolescents with obesity were nearly five times more likely to become adults who were obese than counterparts who were not obese. Further, an estimated 55% of children who are obese will continue to be adolescents who are obese, and 75% to 80% of them will remain adults who are obese. [4]. This prevalence of obesity in childhood and adolescence increases the risk of chronic non-communicable diseases such as hypertension, hyperlipidemia, type 2 diabetes, and atherosclerosis in adults [5].

A crucial causative factor of obesity in children and adolescents is the long-term disequilibrium between energy intake and expenditure [6]. A healthy diet and recommended physical activity (PA) are the best methods for preventing obesity and controlling body weight. PA at recommended levels can reduce body weight, total body fat, and abdominal fat in children and adolescents with obesity [7] and improve body composition [8,9,10]. Additionally, it can modify cardiovascular and metabolic risks [11,12,13], lower inflammatory responses [14,15], and enhance cardiorespiratory fitness [16,17].

PA is a health-enhancing behavior that reduces the risks of being overweight and obese and its adverse cardiovascular and metabolic consequences. The Chinese Physical Activity Guidelines for children and adolescents aged 6 to 17 recommends at least 60 min of daily moderate-to-vigorous (mod-vig) PA, muscle- and bone-building exercise at least three days a week, and reducing sedentary behaviors, including screen time [18]. However, only one-third (29.9% to 34.1%) of Chinese children and adolescents meet the mod-vig PA guidelines [19]. Among those who are overweight and obese, the prevalence of meeting the mod-vig PA guidelines is 15.1% and 10.7%, respectively [19]. Knowing the PA intensity is essential in ensuring the appropriate dose of daily PA and the safety and effectiveness of exercise. PA intensity is defined as 1.6 to 2.9 METs for light intensity, 3.0 to 5.9 METs for moderate intensity, and ≥6.0 METs for vigorous intensity [20]. An MET is the activity metabolic rate divided by a resting metabolic rate. Several Compendiums of Physical Activities are created to identify MET intensities for different age groups, including youth [21], adults [22], and older adults [23]. The Youth Compendium of Physical Activities categorizes MET intensities for 196 PAs for children and adolescents aged 6–9, 10–12, 13–15, and 16–18. However, MET intensities are not stratified by overweight and obesity status. Energy expenditure and the energy cost of walking and running are higher in children and adolescents who are obese than in those who are not obese due to various factors that increase the mechanical costs of movement [24,25]. In a comparison study of energy expenditure (EE) during treadmill walking at the same speeds, the net metabolic cost of walking normalized by body mass was 25% higher in obese than non-obese counterparts due to factors related to body fat and gait mechanics [25].

Few studies identify the energy costs of PA in children and adolescents with obesity. Of those studies published, small sample sizes present challenges in identifying precise METs and EE values for PAs performed by children and adolescents with obesity [26,27]. These factors make it challenging to develop a future Compendium of Physical Activities for children and adolescents who are obese and also lead to inaccuracies in assessing their energy expenditure during physical activity.

Therefore, in this study, we aimed to determine the energy expenditure characteristics of 37 diverse PAs in children and adolescents with obesity and examine the association between obesity and energy expenditure characteristics.

## 2. Methods

### 2.1. Participants

**Figure 1:**
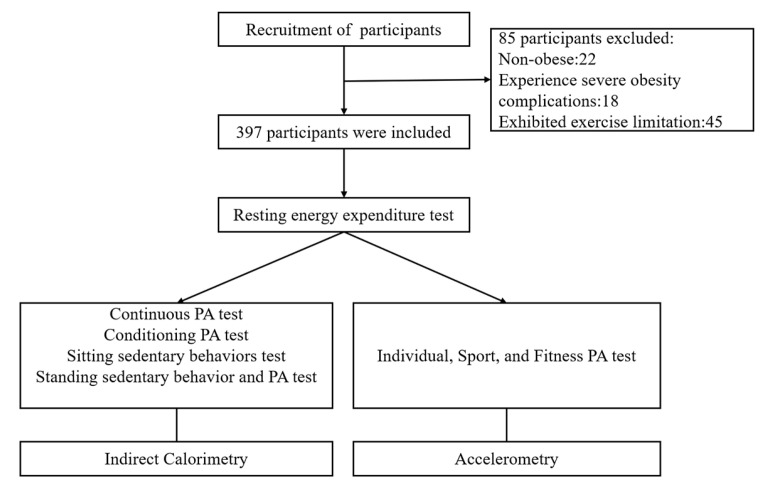
Study flowchart.

Between 2020 and 2023, 397 children and adolescents aged 9–17 with obesity were recruited for this study and completed a series of tests (Figure 1). The inclusion and exclusion criteria are listed in the Supplementary Materials. All participants and their guardians knew of this study and were offered an informed consent form before testing. This study gained the approval of the Human Ethics Committee of Guangzhou Sports University (approval number: 2018LCLL-008).

### 2.2. Anthropometric Indicator Measurements

Standardized height and weight scales (Suhong RGZ-160, Changzhou, China) were used to measure height and weight, which were accurate at 0.1 cm and 0.1 kg, respectively. Height and weight were measured twice and averaged. Based on height and weight, BMI (kg/m^2^) was computed and used as an indicator for the diagnosis of obesity. The diagnosis of obesity is based on BMI cut-off values for different age groups and sexes among Chinese children and adolescents [28].

### 2.3. Bioelectrical Impedance Analysis

Bioelectrical impedance analysis (BIA) determined the participants’ percent body fat, and each participant underwent a body composition test. The body composition analyzer Inbody 370 (InBody, Seoul, Republic of Korea) was used. Body composition measurements were conducted from 7:00 to 8:00 a.m. after ≥10 h of fasting. The participants were instructed to stand with their feet together, holding the electrodes with both hands and then to position themselves with their legs slightly apart. They were asked to raise both arms at a 45° angle from the body and maintain a stable posture during the test.

### 2.4. Indirect Calorimetry

Oxygen uptake (VO_2_) and EE during rest and various commonly performed PAs were tested by indirect calorimetry, which has been widely used in VO_2_ and EE testing studies [29,30]. In the present study, a Cortex Meta Max 3B (Cortex, Leipzig, Germany) portable gas metabolism analyzer was used to monitor VO_2_ and carbon dioxide exhalation (VCO_2_) in real time for each respiration using the MetaSoft Studio 4.6 software. Before the test, the experimenter needed to perform instrument calibration, which included ambient gas calibration (before each test), volumetric calibration (once daily), and standard gas calibration (weekly, with a standard gas mixture of 5% O_2_, 15% CO_2_, and 80% N_2_). Except for Individual, Sport, and Fitness PAs in which EEs were interpolated from accelerometry, the VO_2_ and EE for all individual PAs were measured by indirect calorimetry. During the test, the participants synchronously wore a Polar H10 sensor (Polar Electro Oy, Kempele, Finland) to monitor their heart rate (HR). The validity of the measurement of their HR was confirmed by previous studies, with a correlation coefficient of 0.997 between the results of monitored and electrocardiogram (ECG)-monitored HR.

### 2.5. Heart Rate and Motion Monitoring

A Polar OH1 (Polar Electro Oy, Kempele, Finland) team heart rate (HR) sensor and ActiGraph GT3X+ (ActiGraph, Pensacola, FL, USA) 3-axis motion accelerometer were used to record the participants’ exercise HR and movement acceleration during Individual, Sport, and Fitness PAs, respectively. Before starting the tests, the experimenter initialized the GT3X+ as per the instructions of the manufacturer. Then, the initialized GT3X+ was comfortably fixed to the junction of the midpoint of the participants’ right axillas and the level of the iliac crest via an elastic waistband [31]. In this study, a sampling frequency and interval of 30 Hz and 10 s were used, respectively. The VM_3_, TEE, and MET values of the participants’ PAs were collected through the Actilife 6.13 companion software [32].

### 2.6. Physical Activity Tests

Table 1 lists the 37 PAs performed, divided into five categories (Continuous PAs, Conditioning PAs, Individual, Sport, and Fitness PAs, Sitting Sedentary Behaviors, and Standing Sedentary Behaviors). All PA measurements were scheduled in a fasting state or at least 2 h after a meal to exclude the thermic effect of food in calculating the EE. The participants were asked not to consume coffee or alcoholic beverages for 24 h before testing. PA testing was completed within one week for all participants. See Table S1 for the measurement details of the 37 PA tests.

#### 2.6.1. Resting Energy Expenditure (REE)

The REE test was conducted between 7:30 a.m. and 10:00 a.m., and the participants fasted for at least 10 h prior to the test. Upon arrival at the test site, where the room temperature was maintained at 25–27 °C, the participants remained seated and wore the Meta Max 3B (Cortex, Leipzig, Germany) apparatus for 5–15 min. After their heart rate (HR) stabilized, the participants lay quietly in a supine position for at least 15 min. The test officially began when a steady state was reached. A steady state is defined as a respiratory quotient (RQ) change of less than 5%, oxygen uptake (VO_2_) change of less than 10%, and a minute ventilation change of less than 10% for at least 5 min [33]. An RQ ranging from 0.7 to 1.0 is considered acceptable for the resting energy expenditure test. The 1st–10th min was the adaptation period, and the 11th–15th min was selected to calculate REE and HR_rest_ (resting heart rate).

#### 2.6.2. Exercise Tests: Continuous PAs

##### Field and Treadmill Walking and Running

Ten walking and running tests were performed on a field track (*n* = 5) and on a motorized treadmill (*n* = 5). In the indoor layout of a 10 m × 10 m square sports field, a sign cylinder was placed every 5 m. The participants were required to move along the periphery of the test site, and the speed of movement was controlled through cues. The time intervals of cues were 6, 4.5, 3.6, 3, and 2.57 s (3, 4, 5, 6, and 7 km/h), respectively.

Walking tests were performed at 3, 4, and 5 km/h and running tests were performed at 6 and 7 km/h. Each test lasted 5 min without a break. The 1st–3rd mins of each speed were the adaptation period, and the 4th–5th mins reflected the VO_2_ of the walking and running exercises. See Table S1 for the measurement details of the walking and running tests.

##### Elliptical Trainer

The participants used the ZF-9000 (Shuyoute, Dezhou, China) electromagnetic resistance elliptical trainer to pedal the elliptical trainer at 40 rpm for level 1–5 resistance (corresponding to light effort) (*n* = 5), with each level of resistance lasting for 5 min, and no resting period between tests. The total exercise lasted 25 min, with 1st–3rd mins of each resistance level being the acclimatization period. The data from the 4th–5th min period were selected to reflect the VO_2_ of the exercise. See Table S1 for the measurement details of the elliptical tests.

#### 2.6.3. Conditioning PAs

Conditioning PAs included eight PAs of self-weight and quantitatively loaded conditioning exercises lasting 1 min each with 3 min resting period between PAs. The self-weight-bearing PAs were 1 min squat, 1 min jumping jacks, 1 min walking lunge, and 1 min kneeling push-ups. Quantitively loaded PAs were 1 min dumbbell squat (5 kg load), 1 min dumbbell rowing (5 kg load), 1 min dumbbell press (2.5 kg load), and 1 min bicep curls (2.5 kg load). The data from the last 30 s of exercise were selected to reflect the VO_2_ and energy expenditure [34]. See Supplementary Materials Tables S1 and S2 for the measurement details of the Conditioning PA tests.

#### 2.6.4. Individual, Sport, and Fitness PAs

Seven commonly performed individual, dual sport, and team PAs were selected to identify the estimated EE of various PAs using an ActiGraph GT3X+ (ActiGraph, Pensacola, FL, USA) 3-axis motion accelerometer. The PAs were rhythmic gymnastics, stretching, basketball, circuit training, flag football, badminton, and elliptical trainer (self-selected pace and load). The individuals taking part in this test were recruited voluntarily from campers who had PA sessions scheduled for the week. A professional coach led the PA of each session. Each PA session lasting approximately 30–45 min and data from the sixth minute until one minute before the end of the session were chosen to calculate the average HR and EE for the session. See Tables S1 and S3 for the measurement details.

#### 2.6.5. Sitting Sedentary Behaviors

These four activities were performed in a sitting posture and included writing, reading, listening to music, and playing a video game. Each activity lasted 5 min without a resting period between activities. The final 2 min period of each activity was used to reflect the VO_2_ and EE.

#### 2.6.6. Standing Sedentary Behavior and PAs

These three activities comprised standing, step up and down (with a 20 cm high aerobic step platform, self-paced), and horse stance. The standing quietly and step up and down tests lasted 5 min each, while the horse stance lasted 1 min. There was a 3 min break between each PA. The final 2 min period was used to reflect the VO_2_ of standing quietly and step up and down, with the last 30 s of the horse stance used to measure the VO_2_.

### 2.7. Data Processing and Statistical Analysis

#### 2.7.1. Data Processing

The measured VO_2_, total energy expenditure (TEE), PA energy expenditure (PAEE), METs, resting heart rate (HR_rest_), heart rate for PA (HR_PA_), and percentage of estimated maximum heart rate (% estimated HR_max_) were calculated. Among them, REE and TEE were obtained based on the extraction of VO_2_ (mL/min) and VCO_2_ (mL/min) using the formula of Weir [35]. The specific formulas involved in this study are as follows:

(1) TEE or REE (kcal/min) = 3.9 × VO_2_ (L/min) + 1.1 × VCO_2_ (L/min);
(2) PAEE (kcal/kg/min) = TEE (kcal/kg/min) − REE (kcal/kg/min);
(3) Estimated HR_max_ = 220 − age (year);
(4) % estimated HR_max_ = HR_PA_/estimated HR_max_ × 100%;
(5) METs = Exercise VO_2_ (mL/min)/resting VO_2_ (mL/min) [36].

#### 2.7.2. Statistical Analysis

A data distribution normality test was performed before the statistical analysis. Mean ± standard deviation (Mean ± SD) or median (25–75th percentile) were used to present normally and non-normally distributed data, respectively. Depending on the data distribution, an independent samples *t*-test or Mann–Whitney U test was conducted to compare the differences by sex. Linear regression explored the relationships between age, BMI, percentage BF, and METs, VO_2_, and PAEE. PA intensity was expressed as MET values: sedentary behaviors (1.0–1.5 METs), light-intensity (1.6–2.9 METs), moderate-intensity (3.0–5.9 METs), and vigorous-intensity PA (≥6.0 METs) [20,21,37]. IBM SPSS 24.0 (IBM, Armonk, NY, USA) and GraphPad Prism 9 (GraphPad Software Inc., San Diego, CA, USA) were used for statistical analysis and the graphical plotting of the data. A *p* < 0.05 was accepted as statistically significant.

## 3. Results

### 3.1. Basic Characteristics of the Participants

The participant characteristics are shown in Table 2. In total, 397 children and adolescents with obesity (231 boys and 166 girls) participated in the study. There were significant differences in height, body mass (BM), and BF(%) between boys and girls (*p* < 0.01). The other indicator values were similar between the sexes (*p* > 0.05).

### 3.2. Energy Expenditure and Oxygen Uptake Characteristics of the Resting State

Table 3 presents the resting VO_2_ and EE values for the total sample and by sex. The resting VO_2_ values were higher in obese boys than girls for resting VO_2_ in L/min and mL/kg/min and for REE (*p* < 0.05). Resting heart rate in bpm and % predicted HR_max_ was higher in obese girls than boys (*p* < 0.05).

### 3.3. Energy Expenditure Characteristics of PAs

#### 3.3.1. Energy Expenditure Characteristics of Continuous PAs

The energy cost for children and adolescents with obesity while walking and running at speeds of 3–7 km/h on a field and treadmill, and exercising on an elliptical trainer, for all participants combined is shown in Table 4. Field and treadmill walking at 3 km/h was classified as light intensity (METs < 3.0). Field and treadmill walking at 4 km/h and 5 km/h and running at 6 km/h on a treadmill were classified as moderate intensity (METs 3.0 to 5.9). The remaining PAs (field running at 6 km/h and field and treadmill running at 7 km/h) were classified as vigorous intensity (METs ≥ 6.0). Exercising on an elliptical trainer at 40 rpm during level 1–5 resistance were classified as moderate intensity.

The results of the comparison of the differences between sexes are shown in Table S4.

#### 3.3.2. Energy Expenditure Characteristics of Conditioning PAs

Among the eight self-weight-bearing and quantitative loading PAs (Table 4), the MET values for the self-weight bearing PAs ranged from light (walking lunge, kneeling push-ups) to vigorous intensity (squat and jumping jacks). Among the externally loaded PAs performed for 1 min, the 1 min dumbbell squat and 1 min dumbbell rowing were classified as light intensity, and the 1 min dumbbell press and 1 min bicep curl PAs were classified as sedentary behaviors.

### 3.4. Energy Expenditure Characteristics of Sitting Sedentary Behaviors

Writing, reading, listening to music, and playing a video game were classified as sedentary behaviors (Table 4).

### 3.5. Energy Expenditure Characteristics of Standing Sedentary Behavior and PAs

Standing quietly, the 1 min horse stance, and the step up and down PAs were classified as a sedentary behavior, light-intensity PA, and moderate-intensity PA, respectively (Table 4).

### 3.6. Energy Expenditure Characteristics of Individual, Sport, and Fitness PAs

The TEE, MET values, and mean HR_PA_ of these seven PAs are shown in Table 4. All of these PAs programs except stretching were of moderate intensity for children and adolescents with obesity.

### 3.7. Factors Affecting the Energy Expenditure of PAs

#### 3.7.1. Relationships Between Age, Obesity Degree, and REE

**Figure 2:**
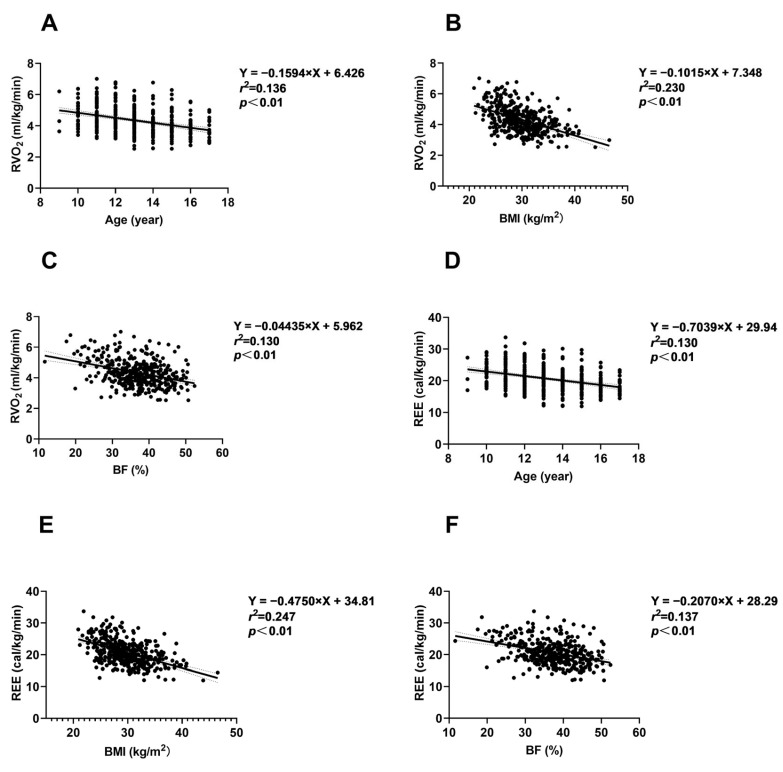
Relationships between age, obesity degree, and resting energy expenditure characteristics. (**A–C**) Relationships between age, BMI, BF, and RVO_2_. (**D–F**) Relationships between age, BMI, BF, and REE. The black dots represent the measured values, the black line represents the fitted line, and the dashed line represents the 95% confidence interval. Abbreviations: BF = body fat; BMI = body mass index; REE = resting energy expenditure; RVO_2_ = resting oxygen uptake.

As shown in Figure 2A,D, resting VO_2_ (mL/kg/min) and REE (kcal/kg/min) decreased with age in children and adolescents aged 9–17 (*p* < 0.01). Increasing degree of obesity was significantly associated with decreasing resting VO_2_ and REE (*p* < 0.01) (Figure 2B,C,E,F).

#### 3.7.2. Relationships Between Age, Obesity Degree, and Energy Expenditure for PAs

**Figure 3:**
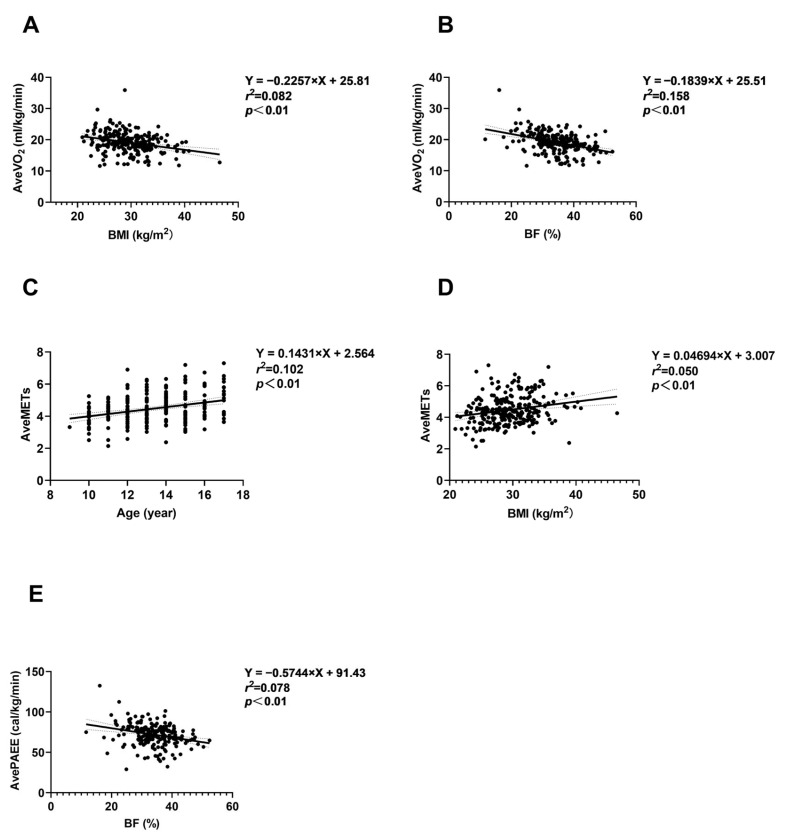
Relationships between age, obesity degree, and field walking and running energy expenditure characteristics. (**A**,**B**) Relationships between BMI, BF, and AveVO_2_. (**C**,**D**) Relationships between age, BMI and AveMETs. (**E**) Relationship between BF and AvePAEE. The black dots represent the measured values, the black line represents the fitted line, and the dashed line represents the 95% confidence interval. Abbreviations: AveMETs = average metabolic equivalent of task; AvePAEE = average physical activity energy expenditure; AveVO_2_ = average oxygen uptake; BF(%) = body fat; BMI = body mass index.

Figure 3A,B show an inverse association between BMI and percentage BF with average VO_2_ (AveVO_2_) during 3–7 km/h field walking and running (*p* < 0.01). Figure 3C,D shows a positive association between age and BMI and the average METs (AveMETs) during 3–7 km/h field walking and running (*p* < 0.01). Figure 3E shows an inverse association between percentage BF and the average PAEE (AvePAEE) during 3–7 km/h field walking and running (*p* < 0.05). The associations between age, BMI, percentage BF, and PA intensity and energy expenditure related to other PAs are presented in Figures S2–S5.

## 4. Discussion

We measured the oxygen cost of 37 PAs to calculate the MET intensity and EE in children and adolescents with obesity. Among these activities, seven were categorized as sedentary behaviors (writing, reading, listening to music, playing a video game, standing quietly, 1 min dumbbell press, and 1 min bicep curls). Eight were classified as light intensity (stretching, walking at 3 km/h in the field and on a treadmill, 1 min dumbbell squat, 1 min dumbbell rowing, 1 min kneeling push-ups, 1 min walking lunge, and 1 min horse stance). Of the mod-vig PAs, 17 were moderate intensity (walking at 4–5 km/h in the field and on a treadmill, running at 6 km/h on a treadmill, using the elliptical trainer at levels 1–5 and with slight effort, step up and down with a 20 cm high aerobic step platform, rhythmic gymnastics, basketball, circuit training, flag football, badminton, and elliptical trainer (self-selected pace, load)), and 5 were vigorous intensity (running at speeds of 7 km/h in the field and on a treadmill, running at a speed of 6 km/h in the field, 1 min squat, and 1 min jumping jacks). The resting VO_2_ and REE were 4.23 mL/kg/min and 0.020 kcal/kg/min, respectively.

Our findings align with previous research indicating that children have higher REE than adults [38,39]. Traditionally, in adults, 1 MET is defined as an oxygen uptake of 3.5 mL/kg/min, which represents the resting metabolic cost [22]. Zhu et al. found that the resting VO_2_ value in Chinese normal-weight adolescents was 6.27 mL/kg/min [40]. Similarly, in a study involving 9–11-year-olds, the resting VO_2_ of normal-weight children was 6.26 mL/kg/min. Another study with children and adolescents in the U.S. found that the resting VO_2_ was 5.1 mL/kg/min in children, also higher than in adults [41]. These findings indicate that the oxygen uptake and energy expenditure associated with 1 MET can vary between children, adolescents, and adults and between obese and normal-weight children and adolescents. Therefore, using resting VO_2_ values for adults or normal-weight children and adolescents to represent 1 MET for children and adolescents who are obese may lead to the underestimation or overestimation of EE in children and adolescents with obesity.

The intensity of exercise directly affects health gains [42,43]. Most PA guidelines for children and adolescents worldwide emphasize that they should engage in 60 min/day of mod-vig PA to maintain health and reduce disease risks [20,44,45]. Except for the 15 PAs classified as sedentary or light intensity, 22 PAs measured in this study were mod-vig intensity. However, the MET values differ for similar PAs performed by children with normal BMI values. For example, in this study’s treadmill walking and running exercises, all speeds, except 3 km/h, were classified as mod-vig intensity. However, the MET values measured were lower for participants than walking and running at similar speeds, as presented in the Youth Compendium of Physical Activities. For instance, the METs of children and adolescents aged 6–18 when walking at 2.5 mph (approximately 4 km/h) range from 3.3 to 3.7 MET_y_ (average, 3.5 MET_y_) across ages 6–18 in the Youth Compendium compared to the 3.09 METs (field) and 3.26 METs (treadmill) measured in this study. The discrepancy is seen with increasing speeds. The Youth Compendium lists MET_y_ values ranging from 6.4 MET_y_ to 8.0 MET_y_ (average, 7.1 MET_y_) for running at 3.5 mph (approximately 5.6 km/h), which is considerably higher than average MET values across ages measured for running at 6.0 km/h in the current study (6.00 METs for field and 5.85 METs for treadmill). Pfeiffer et al. [46] synthesized data from five studies and reported MET values for walking and running exercises at 2 to 5 mph that ranged from 3.56 METs to 8.27 METs in youth aged 6–18. These values are also higher than the MET values recorded for children and adolescents with obesity in this study at similar speeds.

The elliptical trainer is a low-impact PA with less knee stress than running on a treadmill. This PA is particularly beneficial for severely obese individuals or for those with knee discomfort. In this study, participants exercised on an elliptical trainer with five different resistance levels representing easy effort across all intensity levels. The MET values ranged from 3.22 METs to 4.00 METs from levels 1 to 5. No studies report MET intensities for elliptical trainers in youth with which a comparison can be made.

Only a few studies have examined MET and EE in children and adolescents performing self-weight-bearing or resistance exercises. Harrell et al. [47] found that a leg press is a low-intensity PA (2.08–2.90 METs), while another study identified jumping jacks as a high-intensity PA (9.6 METs) [41]. However, these results did not measure the metabolic cost in children and adolescents with obesity. We identified 1 min jumping jacks and 1 min squats as vigorous intensity. The 1 min dumbbell press and 1 min bicep curl activities were found to be sedentary behaviors, and the remaining 1 min PAs were light intensity.

As expected, reading, writing, and listening to music were classified as sedentary behaviors in this study and the Youth Compendium. However, the MET values for these activities were higher in the Youth Compendium (by ~0.18 MET_y_) than measured in this study. Playing video games and standing quietly were categorized as sedentary behaviors, whereas in the Youth Compendium, they were classified as light-intensity PAs. Given these differences, it is important to recognize that using the MET_y_ values from the Youth Compendium or calculating MET values based on normal-weight children and adolescents may overestimate PA intensity and EE in children and adolescents with obesity.

Previous studies have demonstrated decreased in VO_2_ and EE at rest and during PA in children and adolescents as they age [38,47]. Consistent with these studies, we found that in children and adolescents aged 9–17 years with obesity, VO_2_ and EE during both rest and PA decreased with age. However, the MET values from field walking and running exercises and self-weight-bearing/resistance exercises increased with age. Individuals with higher fat mass or body fat percentage generally have lower basal metabolic rates and EE under the same activity status compared to those with a lower fat mass or BF% [48,49,50,51]. This observation may be due to the different mass-specific metabolic rates of skeletal muscle and fat, with the energy metabolism rate of the same mass of skeletal muscle being approximately 4–5 times that of fat mass [52,53]. Previous studies have indicated that the degree of obesity and body composition are considered factors influencing energy expenditure [54,55,56], but their relationship in adolescent populations remains unclear. Therefore, we performed linear regression analysis to examine the relationship between BMI, BF%, and EE characteristics in children and adolescents with obesity. We observed a decreasing trend in VO_2_ and EE at rest and during various types of PA as BMI and BF% increased.

The findings of this study have significant implications for the field. Often used as a reference, the MET_y_ values in the Youth Compendium may not be suitable for direct application to children and adolescents with obesity, as they often overestimate the MET values for PAs. Given the limited research on PA METs in this population, creating a compendium of PAs for children and adolescents with obesity is warranted. The global rise in obesity among children and adolescents underscores the need for accurate PA MET values in children and adolescents with obesity to develop scientifically sound training programs and to assess program effectiveness objectively. Educators can also leverage the new MET values of various PAs to explain the concept of MET, emphasizing the significance of assessing PA intensity and demonstrating how MET values are used to calculate EE. Therefore, further research is needed to expand the available sources of MET values for PAs performed by children and adolescents who are obese.

This study has a unique focus on the energy cost and EE characteristics of Chinese children and adolescents with obesity. It is the first to explore this area with relatively large samples and diverse PA programs. Key indexes of PAs, including MET values, PAEE, VO_2_, HR_PA_, and percent-estimated HR_max_, were used to describe energy expenditure characteristics in this population. The precise data obtained in this study will provide valuable evidence for physical activity guidelines aimed at children and adolescents with obesity, contributing to the enhancement of global physical activity guidelines.

This study also innovatively clarified the relationships between the age, obesity degree, and EE characteristics of children and adolescents with obesity. However, it is important to acknowledge limitations. The number of PA items in the study was relatively small (*n* = 37) compared to the comprehensive list of PA items in the Youth Compendium [21]. The Youth Compendium comprises numerous PA items based on a wide range of published studies, and a primary requirement for its development is a substantial amount of published literature. Currently, there is a scarcity of relevant research focused on children and adolescents who are obese. Despite including only 37 PA items, this study offers a significant advantage in quantity compared to previous research.

Additionally, the recruitment of participants among school-age children and adolescents with obesity was challenging due to the particular characteristics of this population and the academic pressure. This limited the sample size for some activities, and further research is necessary. Nonetheless, to the best of our knowledge, this study remains one of the largest in terms of sample size among studies on energy expenditure in children and adolescents with obesity. 

## 5. Conclusions

The oxygen cost and EE of 37 PAs were measured in Chinese children and adolescents with obesity. Seven were classified as sedentary behaviors, eight were classified as light-intensity PAs, seventeen were classified as moderate-intensity PAs, and five were classified as vigorous-intensity PAs. On average, the MET values were lower than those for similar sedentary behaviors and PAs in the Youth Compendium. This difference may be due, in part, to the lower resting VO_2_ (mL/kg/min) and PAEE (kcal/kg/min) in children and adolescents who are obese than reported for children and adolescents who are normal-weight. Accordingly, MET_y_ values from the Youth Compendium and other studies based on children and adolescents who are normal-weight may not be appropriate for use in children and adolescents with obesity. Our findings offer precise guidance for the physical activity of children and adolescents with obesity and can serve as a reference for developing targeted physical activity guidelines and compendiums. Future research should further diversify the types of PAs measured.

## Figures and Tables

**Table 1:** List of 37 physical activities and sedentary behaviors performed to measure the VO_2_, EE, and computed MET values in children and adolescents with obesity.

Continuous PAs (*n* = 15)	Conditioning PAs (*n* = 8)	Individual, Sport, and Fitness PAs (*n* = 7)
Field walking	Self-weighted	Rhythmic gymnastics
3.0 km/h	Squat	Stretching
4.0 km/h	Jumping jacks	Basketball
5.0 km/h	Walking lunge	Circuit training
Field running	Kneeling push-ups	Flag football
6.0 km/h	Quantitively loaded	Badminton
7.0 km/h	Dumbbell squat (5 kg)	Elliptical trainer (self-selected pace and load)
Treadmill walking	Dumbbell rowing (5 kg)	
3.0 km/h	Dumbbell press (2.5 kg)	
4.0 km/h	Bicep curls (2.5 kg)	
5.0 km/h		
Treadmill running		
6.0 km/h		
7.0 km/h		
Elliptical trainer		
Level 1		
Level 2		
Level 3		
Level 4		
Level 5		
**Sitting Sedentary Behaviors (*n* = 4)**	**Standing Sedentary Behavior and PAs** **(*n* = 3)**	
Writing	Standing quietly	
Reading	Step up and down	
Listening to music	Horse Stance	
Playing video game		

**Table 2:** Descriptive information of the participants.

Indicators	Total (*n* = 397)	Boys (*n* = 231)	Girls (*n* = 166)
Age (year)	13.00 (12.00–15.00)	13.00 (12.00–14.00)	13.00 (12.00–15.00) *
Height (cm)	163.45 ± 9.28	165.06 ± 10.35	161.22 ± 6.97 **
BM (kg)	80.24 ± 16.21	82.65 ± 17.86	76.90 ± 12.90 **
BMI (kg/m^2^)	29.30 (26.85–32.37)	29.70 (26.80–32.60)	29.02 (27.08–31.91)
FM (kg)	29.61 ± 9.00	29.07 ± 9.09	30.30 ± 8.87
BF (%)	36.86 ± 6.85	35.59 ± 6.98	38.68 ± 6.25 **

Note: * *p* < 0.05, ** *p* < 0.01, between sexes; age and BMI are presented as the median and 25th–75th percentiles. Abbreviations: BM = body mass; BMI = body mass index; FM = fat mass; BF = body fat.

**Table 3:** Resting VO_2_ and energy expenditure in children and adolescents with obesity.

Indicators	Total (*n* = 397)	Boys (*n* = 231)	Girls (*n* = 166)
VO_2_ (L/min)	0.34 ± 0.07	0.36 ± 0.07	0.31 ± 0.05 **
VO_2_ (mL/kg/min)	4.23 (3.72–4.84)	4.38 (3.82–5.01)	4.05 (3.59–4.48) **
REE (kcal/kg/min)	0.020 (0.018–0.023)	0.021 (0.018–0.024)	0.019 (0.018–0.022) **
HR_rest_ (bpm)	80 (72–87)	79.00 (71.15–85.00)	81 (73–89) *
% predicted HR_max_	38.53 (34.78–42.01)	37.92 (34.46–41.06)	39.46 (35.67–42.68) **

Note: * *p* < 0.05, ** *p* < 0.01, compared with boys. Abbreviations: bpm = beats per minute; HR_max_ = maximum heart rate; HR_rest_ = resting heart rate.

**Table 4:** Energy expenditure characteristics of 37 PAs in children and adolescents with obesity.

Physical Activity	VO_2_ (L/min)	VO_2_ (mL/kg/min)	MET Values	TEE (kcal/kg/min)	PAEE (kcal/kg/min)	HR_PA_ (bpm)	% Estimated HR_max_
**Continuous PAs**							
Field walking and running							
3 km/h (*n* = 244)	0.94 ± 0.23 ^##^	11.51 (10.23–12.68) ^#^	2.67 (2.35–2.98)	0.055 (0.050–0.060) ^##^	0.033 (0.029–0.038)	118 ± 14 ^##^	57.25 ± 6.76 ^##^
4 km/h (*n* = 244)	1.09 ± 0.24 ^#^	13.64 ± 2.06	3.09 (2.74–3.52)	0.065 ± 0.009	0.044 ± 0.008	126 ± 14	60.97 ± 6.60 ^#^
5 km/h (*n* = 244)	1.39 ± 0.31	17.26 ± 2.64	3.90 (3.47–4.40)	0.082 ± 0.012	0.061 ± 0.011 ^#^	139 ± 15	67.30 ± 7.03
6 km/h (*n* = 232)	2.07 ± 0.52 ^##^	25.65 ± 4.97 ^##^	6.00 ± 1.44	0.127 ± 0.024 ^##^	0.106 ± 0.023 ^##^	168 ± 17	84.09 ± 11.41 ^##^
7 km/h (*n* = 212)	2.37 ± 0.52 ^#^	29.98 ± 5.07	6.70 (5.92–7.70)	0.147 ± 0.025 ^#^	0.126 ± 0.023	180 ± 15	87.20 ± 6.91
Treadmill walking and running							
3 km/h (*n* = 74)	0.86 ± 0.17	11.15 ± 1.56	2.71 ± 0.49	0.053 ± 0.007	0.033 ± 0.006	112 ± 13	54.16 ± 6.18
4 km/h (*n* = 74)	1.04 ± 0.19	13.39 ± 1.76	3.26 ± 0.57	0.064 ± 0.009	0.043 ± 0.007	122 ± 14	59.15 ± 6.84
5 km/h (*n* = 74)	1.36 ± 0.24	17.67 ± 2.26	4.30 ± 0.76	0.084 ± 0.011	0.065 ± 0.010	139 (131–148)	67.23 (63.50–72.01)
6 km/h (*n* = 68)	1.86 ± 0.29	24.25 ± 2.40	5.85 ± 0.92	0.118 ± 0.012	0.098 ± 0.011	165 ± 14	79.47 ± 6.68
7 km/h (*n* = 61)	2.24 ± 0.39	29.13 ± 2.42	7.03 ± 1.05	0.143 ± 0.012	0.123 ± 0.011	181 (171–188)	87.39 (82.73–91.15)
Elliptical Trainer							
Level 1 (*n* = 50)	1.09 ± 0.21	13.25 ± 1.93	3.22 ± 0.55	0.063 ± 0.009	0.043 ± 0.009	128 ± 15	62.04 ± 7.40
Level 2 (*n* = 50)	1.13 ± 0.20	13.79 ± 1.92	3.35 ± 0.54	0.066 ± 0.009	0.046 ± 0.009	132 ± 16	63.83 ± 7.66
Level 3 (*n* = 50)	1.19 ± 0.21	14.60 ± 2.16	3.54 ± 0.58	0.070 ± 0.011	0.050 ± 0.009	135 ± 16	65.33 ± 7.77
Level 4 (*n* = 50)	1.26 ± 0.20	15.40 ± 2.22	3.74 ± 0.61	0.073 ± 0.011	0.054 ± 0.010	139 ± 17	67.10 ± 8.05
Level 5 (*n* = 50)	1.34 ± 0.20	16.47 ± 2.41	4.00 ± 0.70	0.079 ± 0.012	0.059 ± 0.011	144 ± 17	69.50 ± 8.24
**Conditioning PAs**							
1 min squat (*n* = 126)	2.51 (2.00–3.26)	33.68 ± 8.69	8.00 ± 2.37	0.166 (0.137–0.195)	0.146 (0.116–0.174)	151 ± 18	72.87 ± 8.71
1 min jumping jacks (*n* = 131)	2.56 (2.19–3.23)	34.89 ± 8.57	8.14 ± 2.15	0.172 ± 0.043	0.151 ± 0.042	151 (140–161)	73.03 ± 8.45
1 min dumbbell squat (*n* = 32)	0.85 ± 0.17	12.97 ± 2.33	2.91 ± 0.51	0.056 ± 0.009	0.036 ± 0.009	116 ± 12	56.00 ± 5.61
1 min dumbbell press (*n* = 32)	0.40 ± 0.08	6.16 ± 1.25	1.38 ± 0.27	0.027 ± 0.006	0.008 ± 0.005	101 ± 11	48.76 ± 5.45
1 min bicep curls (*n* = 32)	0.41 ± 0.08	6.19 ± 1.07	1.43 ± 0.28	0.027 ± 0.005	0.008 ± 0.005	97 ± 11	46.75 ± 5.47
1 min kneeling push-ups (*n* = 50)	0.70 ± 0.15	8.51 ± 1.35	2.07 ± 0.64	0.041 ± 0.006	0.021 ± 0.006	110 ± 11	53.22 ± 5.67
1 min dumbbell rowing (*n* = 50)	0.60 ± 0.14	7.36 ± 1.39	1.78 ± 0.34	0.035 ± 0.006	0.015 ± 0.006	112 ± 11	53.89 ± 5.38
1 min walking lunge (*n* = 50)	0.90 ± 0.23	10.88 ± 2.08	2.66 ± 0.68	0.052 ± 0.010	0.032 ± 0.010	119 ± 12	57.68 ± 5.76
Individual, Sport, and Fitness PAs ***							
Rhythmic gymnastics (*n* = 25)			3.62 ± 0.96	0.065 ± 0.021		127 ± 15	61.73 ± 7.47
Stretching (*n* = 17)			2.98 ± 0.92	0.037 ± 0.019		116 ± 16	55.97 ± 7.72
Basketball (*n* = 7)			3.28 ± 0.46	0.043 ± 0.008		140 ± 9	67.44 ± 4.48
Circuit training (*n* = 22)			3.52 ± 0.54	0.056 ± 0.012		134 ± 9	64.99 ± 4.53
Flag football (*n* = 16)			4.86 ± 1.01	0.073 ± 0.018		125 ± 15	60.04 ± 7.51
Badminton (*n* = 17)			3.63 ± 0.83	0.059 ± 0.016		134 ± 15	64.95 ± 7.24
Elliptical trainer (self-selected pace, load) (*n* = 16)			3.13 ± 0.55	0.057 ± 0.015		127 ± 7	61.54 ± 3.29
**Sitting sedentary behaviors**							
Writing (*n* = 25)	0.33 ± 0.06	4.57 ± 0.90	1.17 ± 0.15	0.022 ± 0.003	0.003 ± 0.002	84 ± 9	40.71 ± 4.38
Reading (*n* = 16)	0.31 ± 0.04	4.27 ± 0.55	1.15 ± 0.11	0.022 ± 0.003	0.003 ± 0.002	83 ± 9	40.20 ± 4.15
Listening to music (*n* = 13)	0.31 ± 0.06	4.36 ± 0.49	1.12 ± 0.09	0.021 ± 0.002	0.002 (0.001–0.003)	86 ± 6	41.26 ± 2.94
Playing video game (*n* = 46)	0.45 ± 0.10	5.29 ± 0.87	1.25 (1.16–1.49)	0.026 ± 0.004	0.005 (0.004–0.009)	95 ± 11	45.57 ± 5.27
**Standing sedentary behavior and PAs**							
Standing quietly (*n* = 19)	0.35 ± 0.04	4.78 ± 0.70	1.22 ± 0.12	0.020 ± 0.004	0.001 ± 0.002	97 ± 11	46.95 ± 5.13
Step up and down (*n* = 32)	0.92 ± 0.19	12.97 ± 1.56	3.18 ± 0.59	0.060 ± 0.007	0.041 ± 0.008	117 ± 13	56.60 ± 6.11
1 min horse stance (*n* = 47)	0.63 ± 0.15	7.59 ± 1.30	1.85 ± 0.34	0.040 ± 0.006	0.020 ± 0.006	111 ± 11	53.84 ± 5.30

Note: ^#^ *p* < 0.05, ^##^ *p* < 0.01. Comparison with field walking and running activities at the same speed. ***, intensity levels and energy expenditure values were estimated from equations using accelerometry. Comparison of differences using independent samples *t*-test or Mann–Whitney U test. Abbreviations: HR_PA_ = heart rate for PA; HR_max_ = maximum heart rate; MET = metabolic equivalent of task; PAEE = physical activity energy expenditure; TEE = total energy expenditure; VO_2_ = oxygen uptake.

## Data Availability

The data presented in this study are available upon reasonable request from the corresponding author. The data are not publicly available due to privacy reasons.

## Supplementary Materials

The following supporting information can be downloaded at: https://www.mdpi.com/article/10.3390/nu16223966/s1, Table S1: Measurement protocol. Figure S1: Schematic diagram of field walking and running. Figure S2: Relationships between age, obesity degree, and the energy expenditure characteristics of treadmill walking and running. Figure S3: Relationships between age, obesity degree, and energy expenditure characteristics of the elliptical trainer. Figure S4: Relationships between age, obesity degree, and the energy expenditure characteristics of high-frequency conditioning PAs. Figure S5: Relationships between age, obesity degree, and the energy expenditure characteristics of non-high-frequency conditioning PAs. Table S2: Conditioning PA list. Table S3: Individual, Sport, and Fitness PA list. Table S4: Energy expenditure characteristics of 37 PAs in children and adolescents with obesity of different sexes. Table S5: Comparison of the measured and estimated values of PA METs in children and adolescents with obesity. References [57,58,59,60,61] are cited in the supplementary materials.
