# Supplementary material for: Energy Costs of 37 Physical Activities in Chinese Children and Adolescents Aged 9–17 Years with Obesity
Source: Nutrients. 2024 Nov 20;16(22):3966. doi: 10.3390/nu16223966 (PMC11597109; doi:10.3390/nu16223966)
Supplement: Supplementary file 1 [file nutrients-16-03966-s001.zip › nutrients-3318123-supplementary.pdf]

Supplementary material

Methods

Participants

Inclusion criteria were: (1) met the diagnostic criteria for obesity;[1] (2) showed compliance and could actively cooperate with exercise and complete the collection of corresponding indicators. Exclusion criteria were: (1) Suffered from severe obesity complications (confirmed cardiovascular disease, obstructive sleep apnea, musculoskeletal problems, and idiopathic intracranial hypertension, etc.);[2,3] (2) exhibited exercise limitations that prevented them from completing basic exercises.

Physical Activity Test

Table S1. Measurement protocol

Type of physical activity	Physical activity tests	Time (min)	Test requirements	Apparatus
Resting	Resting	15-30	Participants should fast and remain seated quietly for 5 to 15 minutes prior to initiating the test.	Meta Max 3B and Polar OH10
Continuous PAs	3-7 km field walking and running exercise	25	Measurements were conducted at least 2 hours postprandial, and participants performed in 5 minutes of continuous exercise at each speed without intervals.	
	3-7 km treadmill walking and running exercise	25	Measurements were conducted at least 2 hours postprandial, performing 5 minutes of continuous activity at each speed level on a treadmill set to a 0° incline, without intervals between the speeds.	
	Level 1-5 loaded elliptical trainer exercise	25	Measurements were conducted at least 2 hours postprandial, performing 5 minutes of continuous exercise for each loading level, on a treadmill with a 0° incline, without intervals.	
Conditioning PAs, sitting sedentary behaviors, standing	Dumbbell squat, dumbbell press, bicep curl, and step up & down	17	Measurements were conducted at least 2 hours postprandial, 1-min duration for dumbbell squat, dumbbell press, bicep curl, 5-min duration for step up	

sedentary behavior and PAs			& down, and 3-min duration between adjacent PA.	
	Playing video game, kneeling push-ups, dumbbell rowing, walking lunge, horse stance	18	Measurements were conducted at least 2 hours postprandial, 5-min duration for playing video game, 1-min duration for kneeling push-ups, dumbbell rowing, walking lunge, and horse stance. A 3-min duration between adjacent PAs except playing video game	
	Writing, reading, listening to music, and standing	20	5-min duration for each PA ,without intervals.	
	Squat and jumping jacks	5	1-min duration for each PA, with 3min resting between PA	
Individual, Sport, and Fitness PAs	Seven types of Individual sport & fitness PAs training sessions	30-45	Measurements were conducted at least 2 hours postprandial, and the participants were randomly assigned to seven different training sessions without rest during each training session.	Polar OH1 and GT3X+

Individual, Sport, & Fitness PAs HR Monitoring

A Polar OH1 (Polar Electro Oy, Kempele, Finland) team HR sensor recorded the exercise HR of the participants. Its effectiveness in monitoring the HR data of the participants in a variety of exercise programs has been showed by relevant studies.[4] The experimenter attached the Polar OH1 to the participants' left arms during the measurement and subsequently downloaded and analyzed the HR data using the accompanying Polar Team Pro software post-measurement.

Field and Treadmill Walking and Running

Field walking and running exercise at a speed of 3-7 km/h (Figure S1): In the indoor layout of a 10 m × 10 m square sports field, a sign cylinder was placed every 5 m. The participants were required to move along the periphery of the test site, and the speed of movement was controlled through cues. The time intervals of cues were 6, 4.5, 3.6, 3, and 2.57 s (3, 4, 5, 6, and 7 km/h), respectively. The participants were required to arrive at the marker cylinder when cues appeared. To ensure the uniformity of the test, a tester led the test throughout the test. In the meantime, the participants walked and ran around the field at five different speeds, namely

3-7 km/h, with each speed lasting 5 min without rest in between. The 1st-3rd min of each speed level was the adaptation period, and the data of the 4th-5th min smooth period were selected to reflect the VO_2 of the walking and running exercise.

Treadmill walking and running exercise at a speed of 3-7 km/h: The participants performed walking and running exercise on a Precor TRM445 (Precor, Seattle, USA) treadmill with an incline of 0° and at a speed of 3-7 km/h for 5 min at each speed, with an incline of 0° and no rest in between. The 1-3 min of each speed level was the adaptation period, and the data of the 4th-5th min smooth period were selected to reflect the exercise energy expenditure of walking and running exercise.

During the testing of walking and running exercises, the tester used the Borg 15-level (6-20) rating of perceived exertion (RPE) subjective fatigue feeling rating scale[5] to evaluate the fatigue level of the participants to ensure the safety of the experiment. The method of operation was to hold up the RPE scale in the tester's hand at the end of each level of walking and running exercise and ask the participants to self-assess the fatigue feeling level and record corresponding values. During the walking and running exercise, the participants could request to end the experiment at any time if they felt uncomfortable or unable to hold on (exhaustion). The tester had the right to decide to end the experiment when HR > 90% maximum heart rate (Harman), RQ > 1.10, or RPE ≥17.[6]

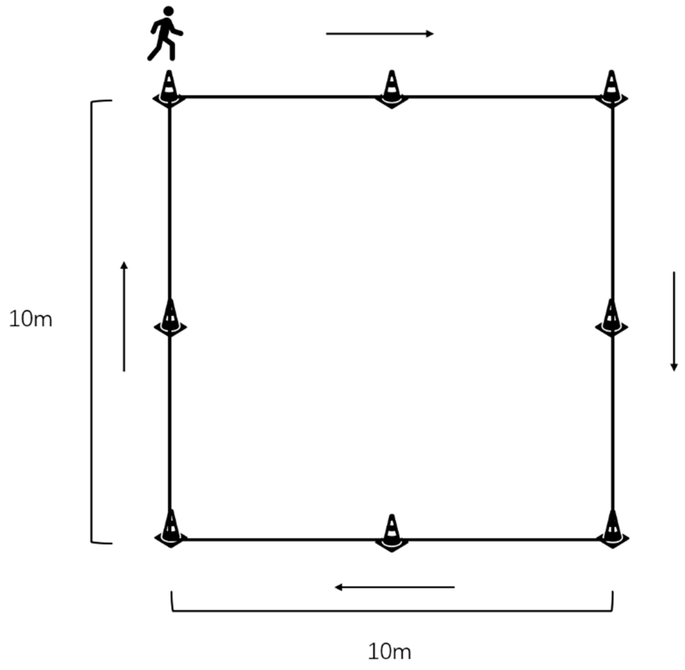

Figure S1. Schematic diagram of field walking and running.

Table S2. Conditioning PAs list.

Physical activity	Description of conditioning PAs	Test requirements
1-min squat	The participants squatted with their knees flexed at an approximate angle of 90 degrees and were required to keep their knees as close to their toes as possible	1 rep/s, 60 rep/min and no extra load
1-min jumping jacks	The participants were required to jump their feet up and out while clapping their hands above their heads. Additionally, they were instructed to keep their legs slightly wider than shoulder-width apart and their hands raised above their heads, near the top of their heads.	
1-min walking lunge	The participants lunged forward with their right and left lower limbs, ensuring their knee joints approximated a 90-degree angle during the lunge.	1 rep/4s and no extra load; right and left lunges both stepping forward was counted as the completion of 1 rep; sitting and standing, kneeling push-ups to complete one up and one down were counted as the completion of one rep
1-min kneeling push-ups	The participants performed push-ups with their knees and hands as pivot points.	
1-min dumbbell squat	The participants were required to squat with their knees flexed to less than 90 degrees and perform maximum squats	1 rep/4s, 15 rep/min and 5 kg extra load
1-min dumbbell rowing	The participants were required to lean forward and straighten their bodies while performing a rowing motion with their right hand, using their back muscles to drive the movement and keeping their elbow joints fixed throughout the exercise.	
1-min dumbbell press	The participants stood upright, holding a dumbbell in their right hand, and pressed it upward from their shoulder to overhead, ensuring their elbow was fully extended at the top.	1 rep/4s, 15 reps/min and 2.5 kg extra load
1-min biceps curl	The participants stood with their right arm fully extended downward, holding a dumbbell in their right hand. Then, they performed a maximal biceps contraction, with the elbow flexion angle remaining less than 90 degrees.	

Table S3. Individual, Sport, and Fitness PAs list.

Physical activity	Description of training content
Rhythmic gymnastics	The instructor selected movements freely and changed them randomly, keeping time with the rhythm of the music.
Stretching	Perform stretching exercises for the core and limb muscles under the coach's guidance.
Basketball	Shooting and dribbling.
Circuit training	Comprised 2 kg weighted medicine ball throws, walking lunge, and plank.
Elliptical trainer(self-selected pace and load)	The participants adjusted the pace and load based on their fatigue levels..
Flag football	Two teams were formed to compete against each other, with participants able to control the pace of the game.
Badminton	A 2 vs. 2 or 1 vs. 1 confrontation, with participants freely controlling the pace of the game.

Results

Table S4. Energy expenditure characteristics of 37 PAs in children and adolescents with obesity of different sexes.

Physical Activity	VO_2（L/min）		VO_2（ml/kg/min）		MET values		TEE（kcal/kg/min）	
	boys	girls	boys	girls	boys	girls	boys	girls
**Continuous PAs**								
Field Walking and Running								
3km/h (n=244)	1.00 ± 0.23##	0.85 ± 0.20**	11.80 (10.53–12.79)	11.28 (10.10–12.33)*	2.67 (2.31–2.92)	2.76 ± 0.60	0.056 (0.050–0.060)##	0.053 (0.048–0.059)*
4 km/h (n=244)	1.15±0.25##	1.02±0.21**	13.69±2.17	13.56±1.89	3.09±0.61	3.18(2.94,3.64)**	0.065(0.059–0.070)	0.065±0.009
5 km/h (n=244)	1.46±0.30#	1.28±0.26**#	17.40±2.81	17.07±2.38	3.82(3.41–4.25)	4.15±0.72#*	0.083±0.012	0.082±0.011
6 km/h (n=232)	2.19±0.55##	1.89±0.42**	26.15±5.44##	24.94±4.12	5.93±1.47	6.11±1.38	0.131±0.026##	0.122±0.021**
7 km/h (n=212)	2.53±0.57#	2.17±0.35**	30.80±5.42	28.91±4.36*	6.83±1.56	6.71(6.07–7.73)	0.151±0.027#	0.143±0.022*
Treadmill Walking and Running								
3km/h (n=74)	0.88±0.18	0.84±0.15	11.36±1.22	10.84±1.95	2.69±0.51	2.74±0.48	0.054±0.006	0.051±0.009
4km/h (n=74)	1.04±0.20	1.03±0.17	13.47±1.47	13.28±2.16	3.19±0.59	3.36±0.52	0.064 ± 0.007	0.063 ± 0.010
5km/h (n=74)	1.36±0.25	1.37±0.21	17.65±2.10	17.69±2.51	4.18±0.79	4.49±0.67	0.085 ± 0.010	0.085 ± 0.012
6km/h (n=68)	1.89±0.33	1.81±0.20	24.40±2.29	23.99±2.60	5.74±0.92	6.03±0.91	0.118 ± 0.011	0.118 ± 0.013
7km/h (n=61)	2.31±0.45	2.13±0.23*	29.55±2.19	28.43±2.68	6.95±1.12	7.17±0.92	0.145 ± 0.011	0.140 ± 0.014
Elliptical Trainer								
Level 1 (n=50)	1.12±0.26	1.05±0.13	13.41±2.18	13.09±1.68	3.12±0.61	3.32±0.48	0.064 ± 0.011	0.062 ± 0.008
Level 2 (n=50)	1.16±0.23	1.10±0.15	13.91±2.09	13.68±1.75	3.23±0.57	3.46±0.49	0.066 ± 0.010	0.065 ± 0.009
Level 3 (n=50)	1.23±0.24	1.16±0.16	14.76±2.36	14.44±1.97	3.43±0.61	3.65±0.53	0.070 ± 0.011	0.069 ± 0.010
Level 4 (n=50)	1.28±0.24	1.23±0.16	15.42±2.39	15.38±2.08	3.58±0.63	3.89±0.56	0.073 ± 0.012	0.074 ± 0.010
Level 5 (n=50)	1.36±0.22	1.32±0.19	16.51±2.62	16.44±2.24	3.83±0.71	4.17±0.65	0.079 ± 0.013	0.079 ± 0.011
**Conditioning PAs**								
1-min squat (n=126)	3.05±0.90	2.24±0.70**	36.19±8.86	30.75±7.55**	8.30±2.45	7.63±2.25*	0.180(0.149–0.222)	0.155±0.039**

1-min jumping jacks (n=131)	3.00±0.81	2.34±0.52**	36.50±9.19	32.97±7.42**	8.25±2.30	8.01±1.97	0.181 ± 0.046	0.162±0.037*
1-min dumbbell squat (n=32)	0.86±0.19	0.83±0.16	13.69±2.00	11.92±2.46	2.95±0.56	2.85±0.44	0.058 ± 0.008	0.052±0.011**
1-min dumbbell press(n=32)	0.42±0.08	0.39±0.08	6.63±1.29	5.61±1.13*	1.45±0.31	1.38±0.26	0.030 ± 0.006	0.026±0.005*
1-min biceps curl (n=32)	0.40±0.07	0.40±0.10	6.41±0.91	5.75±1.27	1.40±0.30	1.39±0.31	0.028 ± 0.004	0.026±0.006*
1-min kneeling push-ups (n=50)	0.74±0.17	0.66±0.12	8.78±1.49	8.23±1.15	2.05±0.41	2.09±0.36	0.042 ± 0.007	0.040 ± 0.005
1-min dumbbell rowing (n=50)	0.64±0.16	0.57±0.10	7.64±1.50	7.08±1.23	1.77±0.34	1.80±0.34	0.037 ± 0.007	0.034 ± 0.006
1-min walking lunge (n=50)	0.92±0.24	0.88±0.23	10.85±1.61	10.95±2.47	2.54±0.54	2.79±0.77	0.052 ± 0.008	0.052 ± 0.011
**Individual, sport, and fitness PAs ***								
Rhythmic gymnastics (n=25)					3.79±1.08	3.45±0.82	0.066±0.026	0.063±0.015
Stretching (n=17)					3.29±0.80	2.71±0.97	0.043±0.016	0.032±0.021
Basketball(n=7)					3.08±0.14	3.37±0.53	0.040±0.006	0.045±0.009
Circuit training (n=22)					3.59±0.60	3.44±0.48	0.057±0.012	0.055±0.012
Flag football (n=16)					3.96±0.87	3.37±0.74	0.066±0.020	0.054±0.011
Badminton (n=17)					3.21±0.64	3.08±0.49	0.057±0.017	0.057±0.014
Elliptical trainer (self-selected pace, load) (n=16)					4.76±1.11	4.99±0.93	0.071±0.021	0.075±0.013
**Sitting sedentary behaviors**								
Writing (n=25)	0.33±0.06	0.32±0.05	4.80±0.61	4.29±0.65	1.21±0.17	1.12±0.10	0.023±0.003	0.020±0.003
Reading (n=16)	0.31±0.04	0.31±0.04	4.43±0.55	4.07±0.53	1.19±0.12	1.09±0.04	0.023±0.003	0.021±0.003
Listening to music (n=13)	0.30±0.06	0.32±0.05	4.47±0.49	4.23±0.51	1.13±0.12	1.11±0.06	0.021±0.002	0.020±0.003
Playing video game (n=46)	0.50±0.11	0.40±0.06	5.66±0.85	4.96±0.77	1.28(1.18–1.60)	1.24(1.14–1.39)	0.027±0.004	0.024±0.004
**Standing sedentary behavior and PAs**								
Standing (n=19)	0.35±0.05	0.34±0.03	4.95±0.70	4.49±0.65	1.22±0.12	1.22±0.12	0.026±0.004	0.023±0.003
Step up & down (n=32)	0.90±0.19	0.96±0.19	13.22±1.45	12.60±1.71	3.12±0.63	3.27±0.55	0.062±0.007	0.060±0.008

Horse stance (n=47)	0.66±0.15	0.59±0.15	7.95±1.38	7.17±1.10	1.85±0.35	1.85±0.33	0.038±0.007	0.034±0.005

Note: *p < 0.05; **p < 0.01, compared with boys; #p < 0.05; ##p < 0.01, comparison of field walking and running PAs with treadmill walking and running PAs at the same speed.

***, Intensity levels and energy expenditure values were estimated from equations using accelerometry. Comparison of differences using independent samples t-test or Mann-Whitney U test.

Abbreviations: HR_{PA} = heart rate for PA; HR_{max} = maximum heart rate; MET = metabolic equivalent of task; PAEE = physical activity energy expenditure; TEE = total energy expenditure; VO_2 = oxygen uptake.

Table S4. Energy expenditure characteristics of 37PAs in children and adolescents with obesity of different sexes(continued).

Physical Activity	PAEE（kcal/kg/min）		HR_{PA}（bpm）		% estimated HR_{max}	
	boys	girls	boys	girls	boys	girls
**Continuous PAs**						
Field Walking and Running						
3 km/h	0.033 (0.029–0.038)	0.033 (0.028–0.038)	117 ± 14##	121 ± 13	56.57 ± 6.95##	58.24 ± 6.22
4 km/h	0.043 ± 0.008	0.045 ± 0.008	123±14#	130±13**	59.72±6.63#	62.74±6.17**
5 km/h	0.059 (0.054–0.067)#	0.062 ± 0.010	136±15	144±14**	65.70±6.92	69.57±6.59**
6 km/h	0.109 ± 0.025##	0.102 ± 0.020*	166±19	170±14*	85.21±13.54##	82.47±7.06**
7 km/h	0.129 ± 0.025	0.123 ± 0.020	179±16	181±12	86.70±7.60	87.93±5.80
Treadmill Walking and Running						

3 km/h	0.033 ± 0.005	0.032 ± 0.008	110±11	116.19±14.45*	52.84±5.27	56.22±6.97*
4 km/h	0.043 ± 0.006	0.044 ± 0.009	119±12	127.73±16.12**	57.45±5.64	61.80±7.76**
5 km/h	0.064 ± 0.009	0.066 ± 0.011	137±14	147.92±17.71**	65.89±6.73	71.58±8.58**
6 km/h	0.098 ± 0.010	0.098 ± 0.012	162±14	168.84±12.59*	78.18±6.76*	81.68±6.06
7 km/h	0.124 ± 0.009	0.121 ± 0.013	179±10	178.39±21.97	86.30±4.99	86.31±10.49
Elliptical Trainer						
Level 1	0.043 ± 0.010	0.043 ± 0.008	125±15	131.84±14.49	60.19±7.46	63.89±6.99
Level 2	0.046 ± 0.009	0.046 ± 0.008	129±16	135.49±15.45	62.01±7.59	65.66±7.44
Level 3	0.050 ± 0.010	0.050 ± 0.009	132±16	138.67±15.97	63.48±7.55	67.19±7.68
Level 4	0.053 ± 0.010	0.054 ± 0.009	135±16	142.92±16.79	64.96±7.58	69.25±8.07
Level 5	0.058 ± 0.012	0.060 ± 0.010	139±16	148.18±17.65	67.20±7.46	71.80±8.48*
**Conditioning PAs**						
1min squat	0.160 (0.127–0.201)	0.135 ± 0.039**	152±19	149±17.00	73.35±9.17	72.26±8.14
1min jumping jacks	0.159 ± 0.044	0.142 ± 0.036*	154(141–161)	148±16.00	74.27(67.80–77.21)	72.01±7.83
1min dumbbell squat	0.038 ± 0.008	0.033 ± 0.009**	118±13	114.16±9.89	56.48±6.29	55.30±4.55
1min dumbbell press	0.009 ± 0.006	0.008 ± 0.005**	104±11	102.51±8.30	49.76±5.04	49.58±3.89
1min biceps curl	0.008 ± 0.006	0.007 ± 0.006**	99±12	99.31±8.99	47.44±5.65	48.07±4.18
1min kneeling push-ups	0.021 ± 0.007	0.020 ± 0.006	105±10	114.92±10.41**	50.73±5.16	55.70±5.13**
1min dumbbell rowing	0.016 ± 0.006	0.015 ± 0.006	110±11	113.19±10.48	52.93±5.58	54.85±5.09
1min walking lunge	0.031 ± 0.008	0.033 ± 0.012	117±10	121.72±12.25	56.32±5.21	58.99±5.96
**Individual sport & fitness PAs *****						
Rhythmic gymnastics			125±16	130±15	60.48±7.58	63.09±7.43
Stretching			118±12	114±19	57.01±6.13	55.03±9.17
Basketball			139±6	139±11	67.54±3.38	67.40±5.21
Circuit training			133±8	135±11	64.39±3.68	65.70±5.513.
Flag football			138±18	131±13	66.71±8.66	63.59±6.09

Badminton			128±4	126±8	61.93±2.32	61.27±3.92
Elliptical trainer (self-selected pace, load)			121±16	129±14	58.53±8.01	61.97±6.89
**Sitting sedentary behaviors**						
Writing	0.004±0.003	0.002±0.002	84±10	85±7	40.20±4.99	41.35±3.57
Reading	0.003±0.002	0.002±0.001	81±9	87±7	38.68±4.34	42.15±3.16
Listening to music	0.003±0.002	0.002±0.001	86±8	85±3	41.10±3.91	41.46±1.51
Playing video game	0.006(0.004–0.012)	0.005(0.003–0.008)	96±10	94±11	46.12±4.99	45.41±5.51
**Standing sedentary behavior and PAs**						
Standing quietly	0.006±0.002	0.005±0.002	100±10	93±11	47.99±5.00	45.17±5.23
Step up & down	0.042±0.008	0.041±0.008	118±12	117±14	56.44±5.99	56.80±6.51
Horse stance	0.017±0.006	0.015±0.005	110±11	113±11	53.01±5.33	54.78±2.24

Note: *$p < 0.05$；**$p < 0.01$, compared with boys； $^{\#}p < 0.05$; $^{\#\#}p < 0.01$, comparison of field walking and running PAs with treadmill walking and running PAs at the same speed. ***, Intensity levels and energy expenditure values were estimated from equations using accelerometry. Comparison of differences using independent samples t-test or Mann-Whitney U test.

Abbreviations: HR_{PA} = heart rate for PA; HR_{max} = maximum heart rate; MET = metabolic equivalent of task; PAEE = physical activity energy expenditure; TEE = total energy expenditure; VO_2 = oxygen uptake.

Table S5. Comparison of the measured and estimated values of PAs METs in children and adolescents with obesity.

Physical Activity	VO_2(ml/kg/min)[a]	Measured METs[a]	Estimated METs[b]	Youth Compendium's METs
**Resting**	4.23	1	1.23**	/
**Continuous PAs**				
Field Walking and Running				
3km/h	11.51	2.67	3.33**	2.8(2.4km/h)*
4 km/h	13.64	3.09	3.92**	3.5(4.0km/h)**
5 km/h	17.26	3.90	4.93**	4.2(4.8km/h)**
6 km/h	25.65	6.00	7.32**	7.2(5.6km/h)**
7 km/h	29.98	6.70	8.53**	7.2(6.4km/h)**
Treadmill Walking and Running				
3 km/h	11.15	2.71	3.19**	2.8(2.4km/h)
4 km/h	13.39	3.26	3.83**	3.5(4.0km/h)**
5 km/h	17.67	4.30	5.05**	4.2(4.8km/h)
6 km/h	24.25	5.85	6.93**	7.2(5.6km/h)**
7 km/h	29.13	7.03	8.32**	7.2(6.4km/h)**
Elliptical Trainer				
Level 1	13.25	3.22	3.79**	
Level 2	13.79	3.35	3.94**	
Level 3	14.60	3.54	4.17**	
Level 4	15.40	3.74	4.40**	
Level 5	16.47	4.00	4.71**	
**Conditioning PAs**				
1-min squat	33.68	8.00	9.48**	
1-min jumping jacks	34.89	8.14	9.63**	

1-min dumbbell squat	12.97	2.91	3.71**	
1-min dumbbell press	6.16	1.38	1.76**	
1-min biceps curl	6.19	1.40	1.77**	
1-min kneeling push-ups	8.51	2.07	2.43**	
1-min dumbbell rowing	7.36	1.78	2.10**	
1-min walking lunge	10.90	2.67	3.12**	
**Sitting sedentary behaviors**				
Writing	4.57	1.17		1.4**
Reading	4.27	1.15		1.2
Listening to music	4.36	1.12		1.3**
Playing video game	5.29	1.25		1.5**
**Standing sedentary behavior and PAs**				
Standing quietly	4.78	1.22		1.5**
Step up & down	12.97	3.18		5.6**
Horse stance	7.59	1.85		

Note: *$p < 0.05$, **$p < 0.01$, compared with the measured values of children and adolescents with obesity in this study; a, experimental results of this study; b, estimated values of PA intensity based on resting VO_2 is 3.5 ml/kg/min. Comparison of differences between measured METs and estimated METs using independent samples t-test or Mann-Whitney U test. Comparison of differences between measured METs and Youth Compendium's METs using one samples t-test

Abbreviations: MET = metabolic equivalent of task; VO_2 = oxygen uptake.

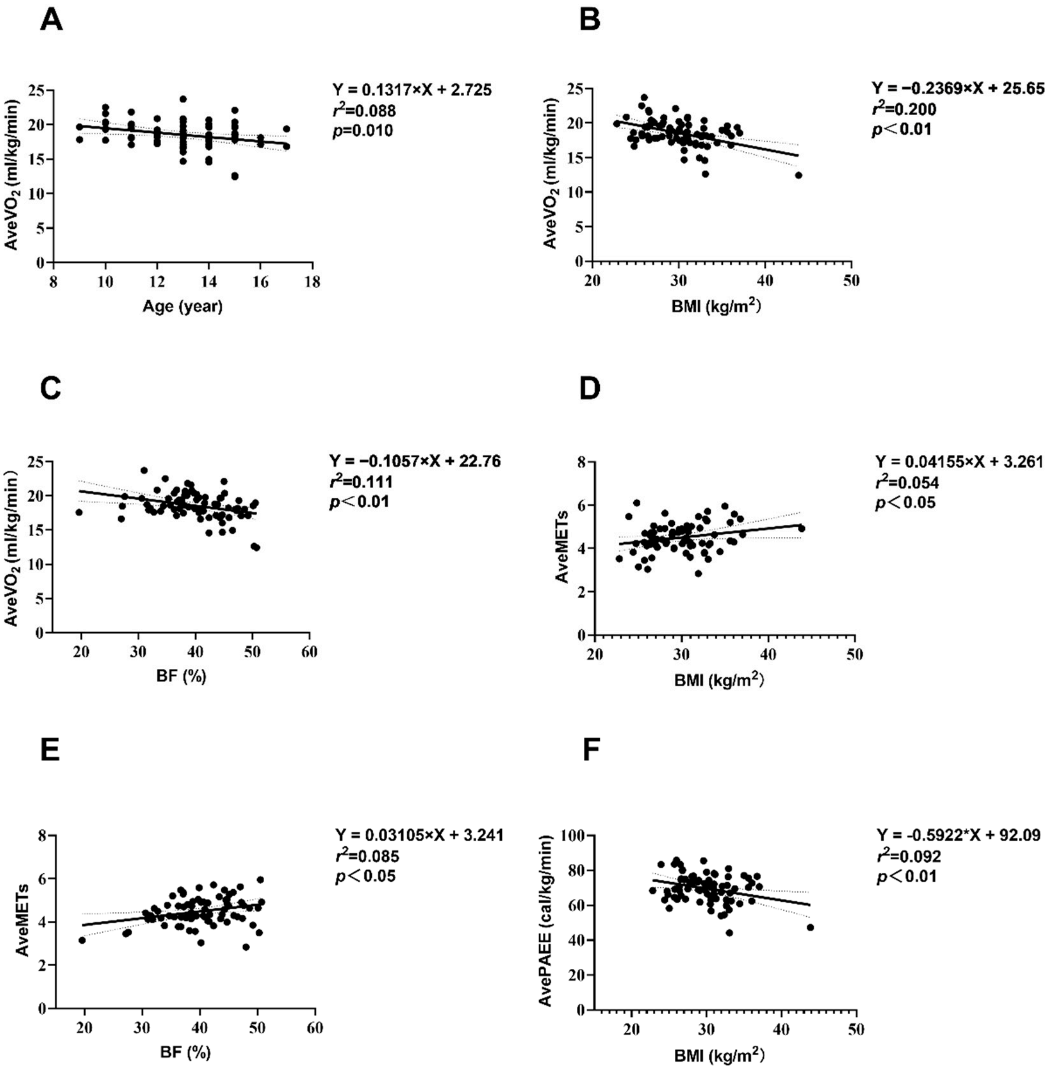

Figure S2. Relationships between age, obesity degree, and the energy expenditure characteristics of treadmill walking and running. (**A,B,C**) Relationships between age, BMI, BF, and AveVO$_2$. (**D,E**) Relationships between age, BMI, BF, and AveMETs. The black dots represent the measured values, the black line represents the fitted line, and the dashed line represents the 95% confidence interval. Abbreviations: AveMETs = average metabolic equivalent of task; AvePAEE = average physical activity energy expenditure; AveVO$_2$ = average oxygen uptake; BMI = body mass index; BF = body fat.

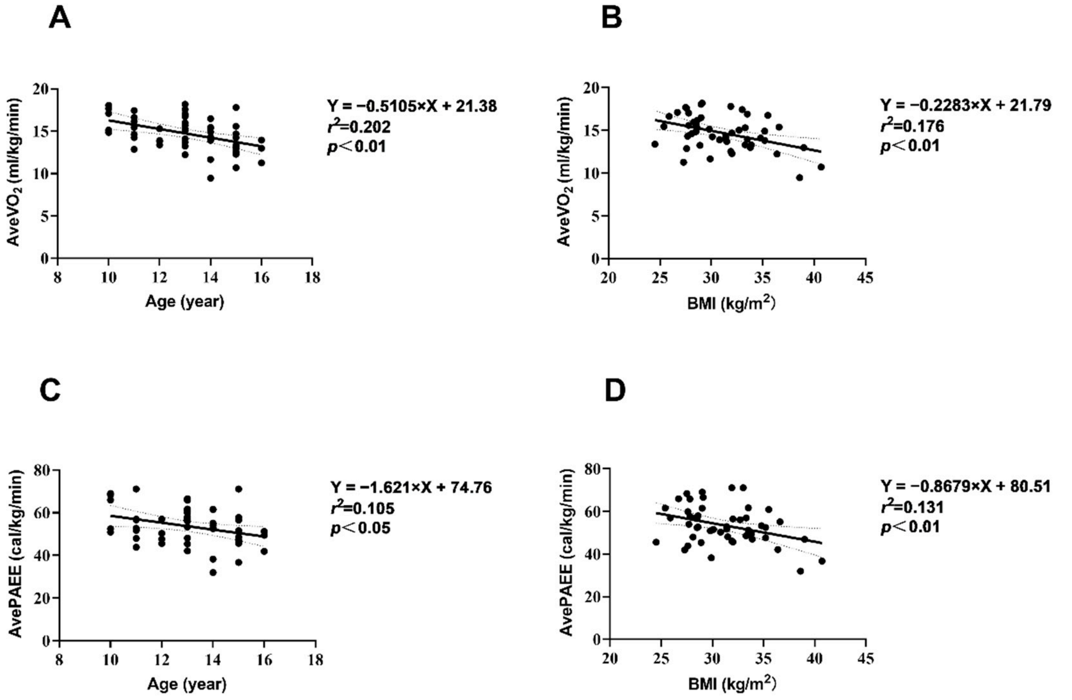

Figure S3. Relationships between age, obesity degree, and energy expenditure characteristics of the elliptical trainer. (**A,B**) Relationships between age, BMI, and AveVO2. (**C,D**) Relationships between age, BMI, and AvePAEE. The black dots represent the measured values, the black line represents the fitted line, and the dashed line represents the 95% confidence interval.

Abbreviations: AvePAEE = average physical activity energy expenditure; AveVO2 = average oxygen uptake.

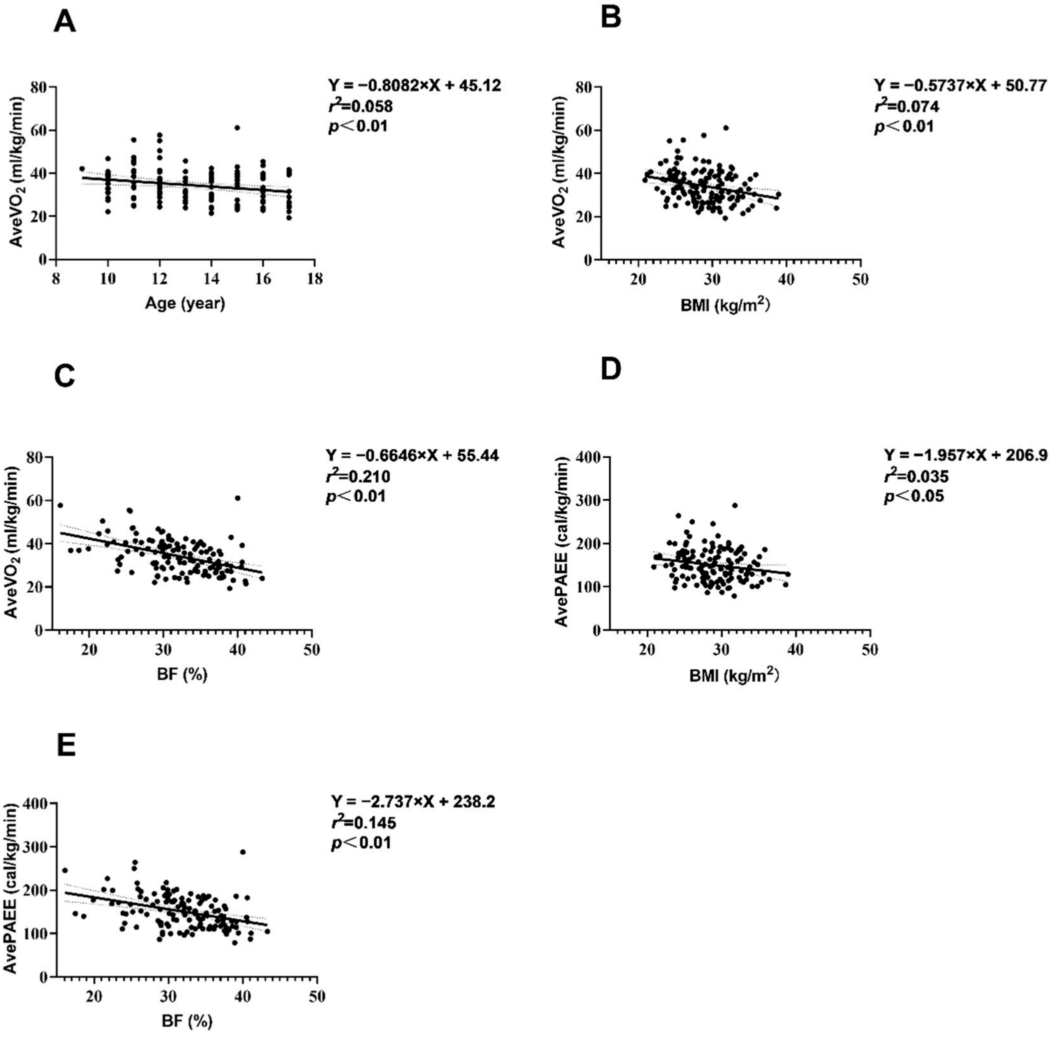

Figure S4. Relationships between age, obesity degree, and the energy expenditure characteristics of high-frequency conditioning PAs. (**A,B,C**) Relationships between age, BMI, BF, and $AveVO_2$. (**D,E**) Relationships between BMI, BF and AvePAEE. The black dots represent the measured values, the black line represents the fitted line, and the dashed line represents the 95% confidence interval.

Note: High-frequency conditioning PAs includes 1min jumping jacks and 1min squat.

Abbreviations: AvePAEE = average physical activity energy expenditure; $AveVO_2$ = average oxygen uptake.

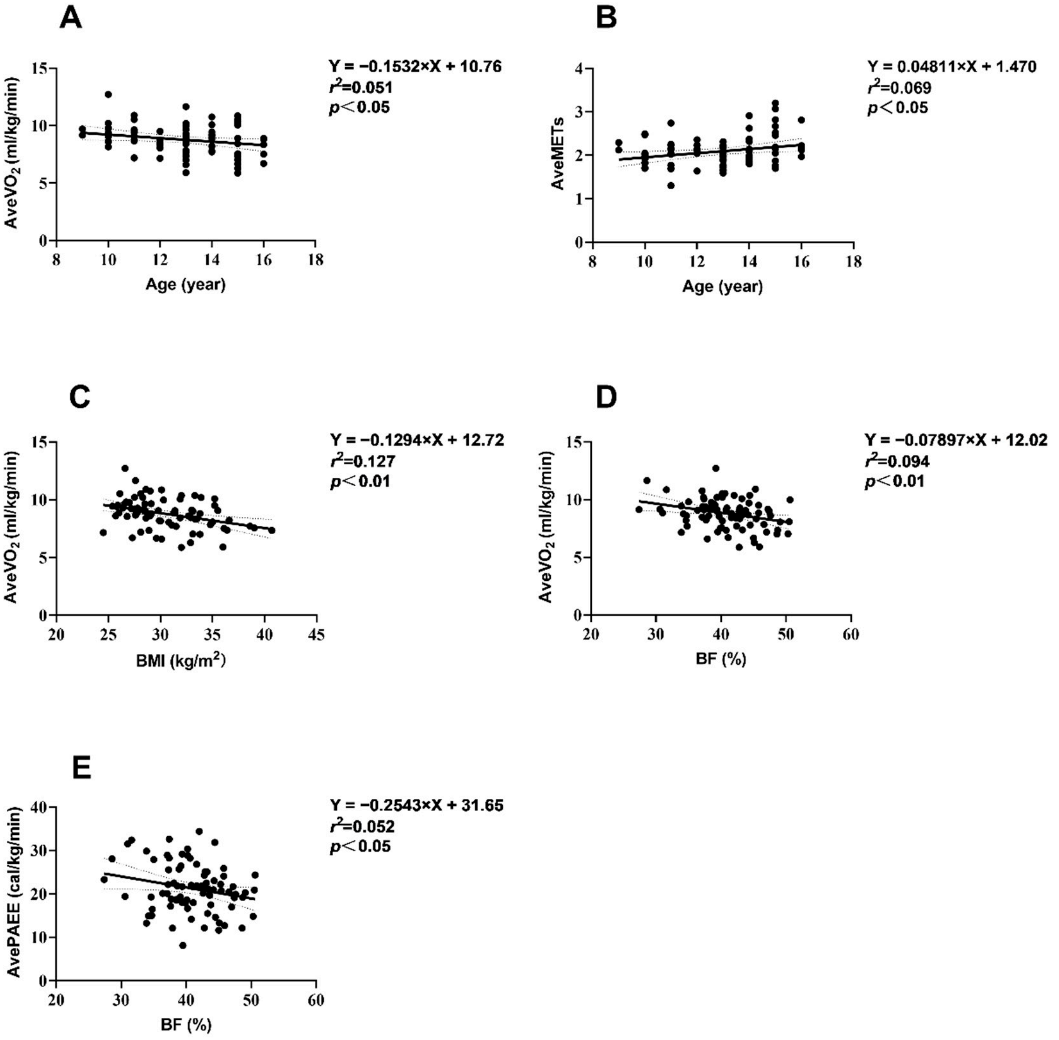

Figure S5. Relationships between age, obesity degree, and the energy expenditure characteristics of non-high-frequency conditioning PAs. (**A,C,D**) Relationships between age, BMI, BF, and AveVO$_2$. (**B**) Relationship between age and AveMETs. (E) Relationship between BF and AvePAEE. The black dots represent the measured values, the black line represents the fitted line, and the dashed line represents the 95% confidence interval.

Note: Non-high-frequency conditioning PAs includes 1min dumbbell squat, 1min dumbbell rowing, 1min walking lunge, 1min dumbbell press, 1min biceps curl, and 1min kneeling push-ups.

Abbreviations: AveMETs = average metabolic equivalent of task; AvePAEE = average physical activity energy expenditure; AveVO$_2$ = average oxygen uptake.

Reference

1. Group of China Obesity Task Force. Body mass index reference norm for screening overweight and obesity in Chinese children and adolescents. *Chin J Epidemiol* **2004**, *25*, 97-102.
2. Ebbeling, C.B.; Pawlak, D.B.; Ludwig, D.S. Childhood obesity: public-health crisis, common sense cure. *Lancet* **2002**, *360*, 473-482, doi:10.1016/s0140-6736(02)09678-2.
3. Kumar, S.; Kelly, A.S. Review of Childhood Obesity: From Epidemiology, Etiology, and Comorbidities to Clinical Assessment and Treatment. *Mayo Clin Proc* **2017**, *92*, 251-265, doi:10.1016/j.mayocp.2016.09.017.
4. Hermand, E.; Cassirame, J.; Ennequin, G.; Hue, O. Validation of a Photoplethysmographic Heart Rate Monitor: Polar OH1. *Int J Sports Med* **2019**, *40*, 462-467, doi:10.1055/a-0875-4033.
5. Borg, G.A. Psychophysical bases of perceived exertion. *Med Sci Sports Exerc* **1982**, *14*, 377-381.
6. Eston, R.G.; Williams, J.G. Reliability of ratings of perceived effort regulation of exercise intensity. *Br J Sports Med* **1988**, *22*, 153-155, doi:10.1136/bjsm.22.4.153.
